# Co-Regulation of Metabolic Genes Is Better Explained by Flux Coupling Than by Network Distance

**DOI:** 10.1371/journal.pcbi.0040026

**Published:** 2008-01-25

**Authors:** Richard A Notebaart, Bas Teusink, Roland J Siezen, Balázs Papp

**Affiliations:** 1 Center for Molecular and Biomolecular Informatics (NCMLS), Radboud University Nijmegen Medical Center, Nijmegen, The Netherlands; 2 NIZO Food Research BV, Ede, The Netherlands; 3 TI Food and Nutrition (WCFS), Wageningen, The Netherlands; 4 Institute of Biochemistry, Biological Research Center, Szeged, Hungary; 5 Faculty of Life Sciences, The University of Manchester, Manchester, United Kingdom; California Institute of Technology, United States of America

## Abstract

To what extent can modes of gene regulation be explained by systems-level properties of metabolic networks? Prior studies on co-regulation of metabolic genes have mainly focused on graph-theoretical features of metabolic networks and demonstrated a decreasing level of co-expression with increasing network distance, a naïve, but widely used, topological index. Others have suggested that static graph representations can poorly capture dynamic functional associations, e.g., in the form of dependence of metabolic fluxes across genes in the network. Here, we systematically tested the relative importance of metabolic flux coupling and network position on gene co-regulation, using a genome-scale metabolic model of *Escherichia coli.* After validating the computational method with empirical data on flux correlations, we confirm that genes coupled by their enzymatic fluxes not only show similar expression patterns, but also share transcriptional regulators and frequently reside in the same operon. In contrast, we demonstrate that network distance per se has relatively minor influence on gene co-regulation. Moreover, the type of flux coupling can explain refined properties of the regulatory network that are ignored by simple graph-theoretical indices. Our results underline the importance of studying functional states of cellular networks to define physiologically relevant associations between genes and should stimulate future developments of novel functional genomic tools.

## Author Summary

Why do certain genes in a biological network show tight transcriptional co-regulation while others are more or less independently regulated? Prior studies showed that the degree of co-regulation between enzymatic genes decreases with their distance in the metabolic network. However, there are fundamental reasons to suspect that network distance is an incomplete descriptor of functional coherence (hence gene co-regulation), and other, biochemically more relevant measures, have been proposed to capture the functional dependencies between enzymes. We systematically examine whether flux coupling, a biochemically sound and computationally tractable measure of functional interaction between reactions, can better explain gene co-regulation than network distance in the metabolisms of Escherichia coli and Saccharomyces cerevisiae. After validating the flux coupling method using published experimental data on in vivo flux correlations (i.e., coherence of reaction usage), we demonstrate that it not only outperforms metabolic network distance in relation to in vivo flux correlations, but also in explaining transcriptional co-regulation and operonic organization. Future functional genomics studies could benefit from the concept of flux coupling by using it as a basis to test the reliability of computationally predicted functional associations.

## Introduction

In recent years, metabolic networks of various species have been reconstructed [1], and several systematic studies addressed the issue of gene regulation in metabolism [2–5]. These studies have revealed important insights into transcriptional regulation by integration of gene co-expression with historically defined modules (e.g., glycolysis) or with graph-theoretical properties of reconstructed networks. Although trends in gene co-regulation with network distance have been reported [3], it remains unexplained how purely graph-theoretical indices of metabolic networks relate to physiologically relevant functional associations. The widely used, but ad hoc, reasoning that when genes are located close to each other on an interaction map, then they will be functionally associated is intuitively reasonable, and, since topological reconstructions are widely available for many species (e.g., KEGG), it forms the basis for many validations of predicted functional associations in cellular networks [3,6–8]. However, there are good reasons to suspect that metabolic network distance per se does not necessarily indicate whether two reactions are used coherently in functional states of the network. For example, all enzymes within a linear pathway might be strongly associated in their function irrespective of their network distances (though their temporal activation patterns can correlate with distance [9]). Moreover, erroneous predictions of functional associations might arise as paths defined on a metabolic connectivity graph do not necessarily correspond to biochemically relevant pathways [10].

**Figure 1:**
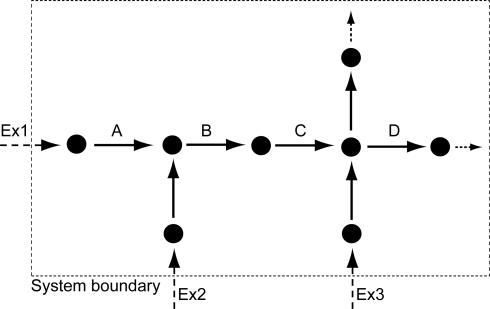
A Hypothetical Network with Metabolites (Nodes), Reactions (Arrows), and Exchange Reactions (Ex) with the Environment Indicated are three types of flux coupling between reactions that are located at distance 1 (directly connected by one node): i) A-B: directionally coupled, ii) B-C: fully coupled, and iii) C-D: uncoupled.

Since the functional state (phenotype) of metabolic networks is best represented by the actual flux distribution [11,12], one might expect that the correlation between reaction fluxes across network states would provide a sound and biochemically relevant measure of functional dependence between enzyme-encoding genes [13]. Therefore, we hypothesized that dynamic functional associations (i.e., correlations) between fluxes, rather than static topological properties of a metabolic network, could capture true functional associations between genes and consequently would provide refined insights into the modes of transcriptional regulation of metabolism. Recently, computationally tractable frameworks have been developed to determine genome-scale functional associations between metabolic genes on the basis of their coherent use of reactions (also referred to as “correlated reaction sets” or “flux coupling”, see Figure 1) [13–16]. Prior studies initialized the integration of gene regulation with flux coupling and concluded that genes with correlated reactions often show signs of co-regulation [14,17–19]. However, these studies did not explore the regulatory consequences of the differences in the degree of flux coupling. Moreover, it remains unknown to what extent flux dependencies relate to graph-theoretical properties of metabolic networks with respect to gene regulation.

In this study we therefore systematically investigated to what extent (degrees of) flux coupling and network distance, a simple and widely used topological index, relate to co-regulation. Although it might seem intuitively likely that reactions are flux coupled at shorter distances, it is easy to imagine situations where even neighboring reactions carry uncorrelated fluxes (see Figure 1); hence it is important to quantitatively assess the contribution of each factor to gene co-regulation. The well-characterized metabolic [20] and gene-regulatory networks [21] of Escherichia coli make it an ideal organism to address these issues. Therefore, we primarily aimed at relating network distance and flux coupling in the metabolic network to the transcriptional regulation of the associated genes in E. coli. In addition, to confirm the generality of our findings, we extended our study to Saccharomyces cerevisiae. Our results demonstrate the importance of flux coupling, rather than network distance, as a better determinant of metabolic gene co-regulation.

## Results/Discussion

### Flux Coupling Captures Physiologically Relevant Functional Associations

To predict reaction sets that appear together in functional states of the network, we performed flux coupling analysis [14] on a genome-scale reconstruction of E. coli metabolism [20]. This procedure identifies coupled biochemical reactions in steady-state flux distributions of the network, given a set of environmental constraints (Methods). Metabolic gene pairs were categorized into three different groups: i) *fully coupled*: non-zero flux for one reaction implies a fixed (non-zero) flux for the other reaction and vice versa, ii) *directionally coupled*: the activity of one reaction implies the activity of the other, but not necessarily the reverse. Thus, these reactions are clearly not independent, but may not always operate together (i.e., the flux of one reaction can be zero while the other carries a flux), and iii) *uncoupled*: reactions whose flux ratios can take up any values, hence can operate independently [14].

**Figure 2:**
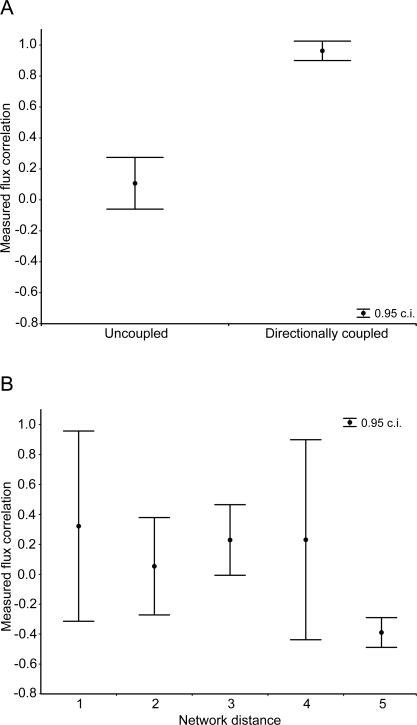
The Average Level of Empirically Determined Flux Correlations for Different Flux Coupling Types (A) and at Different Network Distances (B)

Although phylogenetic [19] and metabolome [22] studies suggest that in silico predicted flux coupling relationships have strong physiological and evolutionary relevance, it remains unexamined how well this procedure can explain in vivo flux correlations. For example, is directional coupling a physiologically relevant category in the sense that these reactions show some, but not perfect flux correlations? An experimental study enabled us to calculate flux correlations between 120 reaction pairs over six conditions in the central carbon metabolism of E. coli [23]. Although none of these reaction pairs were fully coupled, we found a marked difference between the two other coupling groups: directionally coupled reaction pairs had, on average, much higher empirical flux correlations than uncoupled ones (Wilcox robust analysis of variance, ANOVA, *p* < 10^−14^, Figure 2A, see Methods).

In contrast to the association between flux coupling and in vivo flux correlations, we found no clear evidence for such an association for network distance (see Methods): pairs up to a distance of four showed no difference in flux correlation (*p* = 0.77, Figure 2B), and only pairs separated by five metabolites showed a drop in flux correlation (Wilcox multiple pairwise comparison, *p* < 0.05, see Methods).

### Operonic Organization Correlates with Both Flux Coupling and Network Distance

**Figure 3:**
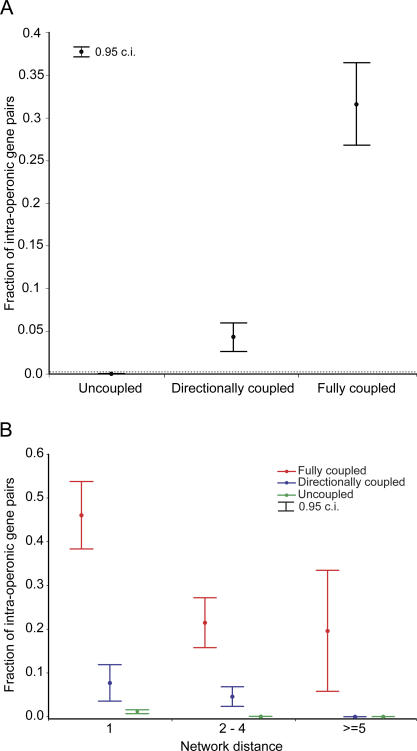
The Effect of Flux Coupling and Network Distance on Operonic Organization in E. coli (A) The fraction of intra-operonic gene pairs correlates with the type of flux coupling. The dashed baseline indicates the fraction of intra-operonic gene pairs expected by chance. (B) The effect of flux coupling on the fraction of intra-operonic gene pairs in different network distance groups: χ^2^_d=1_ = 715.3, χ^2^_d=2,3,4_ = 5347.3, χ^2^_d≥5_ = 5022.3, d.f. = 2, and *p* < 10^−155^.

To measure and compare the extent of co-regulation between the types of flux coupling, we calculated the frequency of gene pairs that are part of the same operon (referred to as intra-operonic) as it represents a clear measure of co-regulation. The comparison revealed an association between the type of flux coupling and the likelihood of being intra-operonic (χ^2^ = 20489.6, d.f. = 2, *p* ≈ 0, Figure 3A). Thus, genes with complete correlation in flux behavior undergo more frequently precise co-regulation. Directionally coupled gene pairs do not necessarily operate together at all times, and, indeed, we find that these pairs less frequently reside in the same operon.

We extended the analysis by categorizing, for each coupling type, gene pairs into three network distance groups: i) distance 1 (direct neighbors); ii) distance 2, 3, and 4 (moderately close); and iii) distance ≥5 (note: the average distance is ∼4.8 in the network). When considering each distance group individually, we still found a significant association between flux coupling and operonic organization at any distance on the metabolic graph (Figure 3B). Moreover, the strength of association, as expressed by Cramer's V, illustrates the importance of flux coupling even when the genes are direct neighbors in the network (V_d=1_ = 0.54, V_d=2,3,4_ = 0.35 and V_d≥5_ = 0.32, where V scales from zero to one). Having demonstrated the importance of flux coupling when controlling for network distance, we next asked if distance has an independent effect by testing the association between operonic organization and distance for each specific type of flux coupling. Although we found a statistically significant association for fully coupled pairs (χ^2^ = 27.3, d.f. = 2, *p* < 10^−5^, Figure 3B), the strength of the effect (V = 0.26) is lower compared to those observed for flux coupling. Moreover, no association was detected in the group of directionally coupled pairs, and the association was weak, though statistically significant, for uncoupled ones (Table S1).

How to explain the correlation between network distance and operonic organization for fully coupled gene pairs? The organization of genes into operons is an ongoing evolutionary process with chance events playing potentially important roles, and therefore the composition of operons might not be optimal [24]. However, non-optimal operonic composition does not automatically imply a negative correlation between the network distance of a gene pair and the probability of both genes residing in the same operon. Thus, although any individual functionally related gene pair might be located in distinct operons simply by chance, our observation that separation into distinct operons depends on metabolic network distance suggests that operonic composition is shaped by selective forces as well. Intuitively, one might argue that at larger distances both genes might simply not fit into the same operon. To explore this possibility, we repeated our analysis with only those fully coupled pairs that are encoded in operons with “non-limiting” sizes. “Non-limiting” operons are defined as those operons that have a size large enough to contain all genes on the shortest path from one gene to the other (e.g., the minimum size of “non-limiting” operons for gene pairs at network distance 2 is three genes). None of the 25 gene pairs that are located in different operons and at a large network distance (i.e., ≥5) were in “non-limiting” operons (Table S2 and S3), hinting at the possibility that limits on operon size might play a role.

However, within the set of gene pairs in “non-limiting” operons, we still see an association between network distance and operonic organization for pairs at distance <5 (Table S3). Thus, structural constraints do not fully explain the separation of genes into different operons at shorter network distances. We speculate that a more likely explanation is that partition of within-pathway genes into multiple operons could allow the temporal fine-tuning of expression patterns in a way that enzymes are not synthesized before needed within a pathway [25]. Such a “just-in-time” transcription program has been predicted to be optimal when the system needs to reach production objectives with minimal total enzyme synthesis [26] and has been supported by experimental studies on amino-acid biosynthetic enzymes [9].

### Transcription Factor Binding Similarity Correlates with Flux Coupling

**Figure 4:**
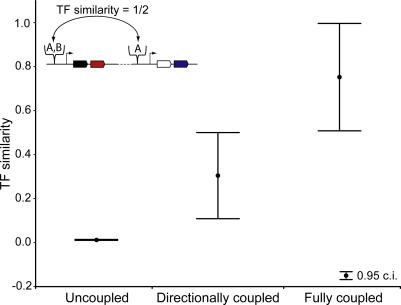
Transcription Factor (TF) Similarity Correlates with the Type of Flux Coupling

As transcription factors (TF) play an important role in the regulation of gene expression, we compared TF co-regulation between the different flux coupling types. We quantified the overlap in TFs upstream of operon pairs (TF similarity) as the number of shared TFs relative to the total number of involved TFs (Methods). As intra-operonic gene pairs show co-regulation by definition, we specifically studied those gene pairs that are encoded in different operons (i.e., inter-operonic) and are controlled by at least one known TF. Moreover, only those operon pairs were selected that contain inter-operonic gene pairs with the same type of flux coupling. We found that fully coupled operon pairs have, on average, higher TF similarities compared to both directionally and uncoupled ones (Wilcox multiple pairwise comparison, *p* < 10^−2^, Figure 4). Uncoupled operon pairs show extremely low TF similarities, which confirm the expectation that it would be irrelevant to co-regulate genes without a functional association.

### mRNA-Level Co-Expression Can Be Better Explained by Flux Coupling Than by Network Distance

It has previously been reported that the level of co-expression decreases with increasing network distances [3]. However, given the evidence that the degree of TF-binding similarity correlates with flux coupling, this observation might be intuitively explained by the possibility that uncoupled gene pairs have higher network distances than coupled ones. Uncoupled (inter-operonic) gene pairs are indeed at larger network distances compared to flux coupled pairs (Figure S1A). To further investigate whether the association between mRNA-level co-expression and network distance might be indirect, we analyzed a large-scale gene expression dataset collected over a variety of conditions [24]. Confirming the finding of Kharchenko et al. (2005) in yeast, we found a significant association between co-expression and network distance in E. coli (Wilcox robust ANOVA, *p* ≈ 0). However, the degree of co-expression was also associated with flux coupling (*p* < 10^−14^, Figure S1B), a finding not unexpected based on the differences in TF similarities between the different types of flux coupling.

**Figure 5:**
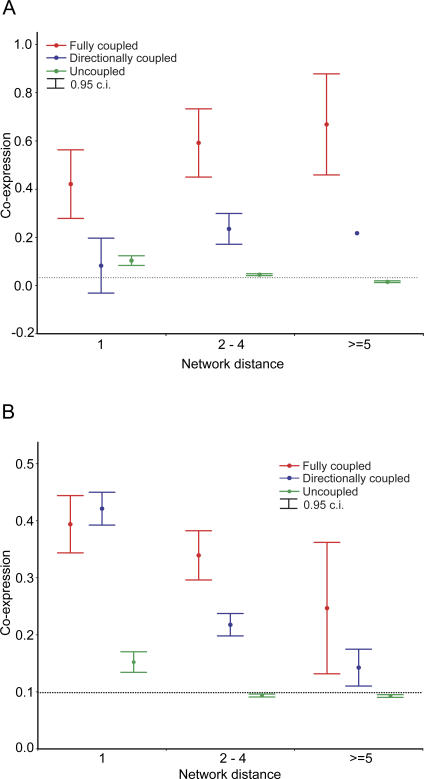
The Effect of Flux Coupling and Network Distance on Co-Expression for E. coli (A) and S. cerevisiae (B) (A) The dashed baseline indicates the degree of co-expression between random gene pairs. The confidence interval of directionally coupled pairs at d ≥ 5 is absent, as it contains too few data points (*n* = 2) for reliable calculation. (B) Relative variance components (i.e., the fraction of total variance in co-expression explained by coupling and distance) were estimated by a general linear model where both flux coupling and distance were treated as random effects in an unbalanced factorial ANOVA design. Expected means squares were used for the estimation (Statistica 6.0, Statsoft). Flux coupling and network distance explain 16.8% and 7.3% of the variance in co-expression, respectively (interaction between the two factors explains 3.7%). A maximum likelihood estimation of variance components gave very similar results (coupling: 14%, distance: 7.1%, and interaction: 3.8%, Statistica 6.0, Statsoft). Note that the average network distance is ∼4.5.

To unveil which factor (i.e., network distance or flux coupling) is the main determinant of co-expression between metabolic genes, we performed a two-way robust ANOVA [27]. We found that while flux coupling is a significant main effect (*p* < 0.003), the effect of network distance is not (*p* = 0.244), and there is an interaction between these two factors (*p* = 0.003) (Figure 5A). Apparently, the interaction term arises because the degree of co-expression increases with network distance for flux coupled gene pairs (*p* < 10^−4^), but decreases for uncoupled pairs (*p* ≈ 0). Hence, network distance does not explain transcript-level co-expression for inter-operonic flux coupled genes in E. coli, and even for uncoupled genes it predicts only weak co-expression for those located close to each other on the metabolic map: uncoupled neighboring (d = 1) gene pairs have an average co-expression of 0.106, which is only slightly, albeit statistically significantly, higher than the 0.039 observed for random pairs (see baseline in Figure 5A). The idea that considering flux coupling relationships improves the discrimination of gene sets with different levels of co-expression is further exemplified by our observation that although fully and directionally coupled gene pairs do not differ in terms of overall network distance (*p* = 0.9, Figure S1A), they differ in co-expression (Figure S1B) and TF similarity (Figure 4, *p* < 10^−2^). Thus, the type of flux coupling can capture differences in the degree of gene co-regulation that are ignored by network distance.

To confirm the above finding on the relatively minor effect of network distance compared to flux coupling, we also examined mRNA-level co-expression of metabolic genes in S. cerevisiae using a high-quality metabolic reconstruction [28] and a large set of microarray data [2] (Methods). Our analysis showed that both flux coupling and network distance are associated with co-expression (two-way ANOVA, *p* < 10^−11^), but flux coupling explains approximately twice as much of the variance in co-expression than network distance (see Figure 5B for details).

In summary, our results illustrate that modes of gene co-regulation can be better explained by a biochemically well-grounded flux correlation based measure (flux coupling), than by network distance, even though distance was calculated by excluding highly connected nodes to minimize artificial shortcuts. Network distance, although widely applied, is by no means the only possible topological measure, and therefore further studies should address whether more sophisticated and more robust graph-theoretical measures could provide refined insights into gene co-regulation.

Furthermore, it should be noted that changes in fluxes are not necessarily caused by changes only at the transcriptional level. Although concerted changes in enzyme levels through transcription may in theory improve metabolite homeostasis during large flux changes [29], experimental studies show that flux changes can arise as a result of specific types of regulation on each individual enzyme in the pathway, (e.g., on the level of metabolite concentrations or on the level of transcription, translation, posttranslational modifications, protein degradation, etc.) [30]. This explains that even for fully coupled gene pairs no strict correlation with transcriptional co-regulation is observed, or could be expected.

Our work has important implications for comparative genomics and gene function predictions. Since metabolic networks are based on solid biochemical knowledge and are the best-characterized biological networks available for numerous species, the present work paves the way for improved gene association studies in the future. In particular, the concept of flux coupling could form the basis to test the reliability of predicted functional interactions by genomic context or high-throughput functional genomics data. Since benchmarking of predicted gene associations (i.e., set of true-positives) relies in many studies on topological properties of pathways and networks (e.g., being associated to the same KEGG map) [7], we expect that considering flux coupling would increase the quality of benchmarks and, as a result, prediction accuracy. In a similar vein, the computational prediction of operons could be improved by using flux coupling information instead of historically defined pathway classifications [31]. One potential difficulty in applying flux coupling for functional genomics is that this approach requires a high-quality, extensively curated reconstruction amenable to stoichiometric modeling [32]. In contrast, topological analyses can be applied to networks of lower accuracies, hence to a wider range of organisms. However, the development of improved functional genomic tools with flux coupling should certainly become feasible given the rapidly increasing number of genome-scale metabolic reconstructions and the availability of constraint-based methods to define flux correlations [1,16].

## Materials and Methods

### Flux coupling analysis.

To analyze functional (physiological) associations between genes within the genome-scale metabolic network of E. coli K12 (*i*JR904 GSM/GPR) [20], we applied the previously developed flux coupling finder procedure (see Dataset S1) [14]. This constraint-based modeling approach relies on minimization and maximization of the flux ratios to determine the extent of dependency between any two reactions within the network given mass-balance constraints and boundary constraints (exchange fluxes with the environment). In general, the flux through one of the two reactions is fixed by a unit value while the flux through the other reaction is maximized and minimized (allowing for linear optimization, see Burgard et al. for details).

We distinguished three main types of flux coupling relationships between reaction pairs (see also Figure 1): i) *fully coupled*: the activity of one reaction fixes the activity of the other and vice versa (i.e., complete correlation by equal minimum and maximum flux ratios); ii) *directionally coupled*: the activity of one reaction implies the activity of the other, but not necessarily the reverse—these reactions are not independent, but may not always operate together (i.e., the flux through one reaction can be zero while the other carries a flux); and iii) *uncoupled*: the activity of one reaction does not imply the activity of the other and vice versa (i.e., their flux ratio can vary from zero to infinity, indicating that the reactions are not (likely) to operate together). Calculations were run without assuming a constant biomass composition to avoid coupling of a large set of fluxes to the biomass reaction (thus all biomass components were allowed to be drained independently of one another) [14]. Coupled reaction pairs were identified under a condition where all external nutrients were allowed for uptake and secretion (i.e., fewest constraints) except for the case where flux coupling was compared to empirical flux correlations. In the latter case a minimal glucose medium was simulated to mimic the experimental settings of Emmerling et al. [23] where fluxes were measured in a wild-type and a mutant E. coli strain under two carbon-limited and one nitrogen-limited growth conditions, corresponding to six experimental setups (note: the same set of nutrients were available for uptake in all six conditions).

Duplicated genes or isoenzymes can give rise to ambiguous relationships between genes and reactions when considering regulatory information. For example, duplicates might be differentially regulated, although their gene products have the same molecular function (i.e., catalysis of the same reaction). We therefore considered reaction pairs that are not associated to isoenzymes to achieve optimal sensitivity for analyzing gene co-regulation among different flux coupling types (and network distance, see below). Furthermore, multiple reaction pairs correspond to the same gene pair when a single gene is associated to more than one reaction. In those cases, we investigated flux coupling of all reaction pairs, but we assigned one type of coupling to the gene pair in the following hierarchical order: fully, directionally, and uncoupled (e.g., if one of the reaction pairs was fully coupled, we considered the corresponding gene pair to be fully coupled irrespective of the other associated reaction pairs).

A similar procedure was applied to the iLL672 reconstruction of the yeast metabolic network [28] to identify flux coupled gene pairs in S. cerevisiae (Dataset S1), with the main difference that we also found partially coupled gene pairs in this network. Partial coupling can be considered as a form of coherent reaction usage with the activity of one reaction implying the activity of the other and vice versa (without, however, a fixed flux ratio between the two reactions) [14]. We therefore grouped fully and partially coupled pairs.

### Network distance.

In order to calculate the network distance between genes within the genome-scale metabolic networks of E. coli and S. cerevisiae, we represented the networks as connectivity graphs consisting of nodes (metabolites) and edges (reactions). Subsequently, we calculated the network distance between any two reactions in the network by a shortest path algorithm based on the connectivity of the nodes. In such a way the distance is defined as the minimal number of metabolites that separates any two reactions in the network. Moreover, information on reversibility and irreversibility of reactions was considered in calculating the shortest paths. Nevertheless, we note that treating all reactions as reversible in order to minimize the number of reaction pairs that are unreachable gives qualitatively the same results (see Tables S4–S7).

The existence of highly connected nodes (such as cofactors) can cause artificial shortcuts in the paths, resulting in biochemically infeasible paths. To increase functional relevance of the network distance, we removed the most highly connected nodes, including ATP, ADP, AMP, CO_2_, CoA, glutamate, H, NAD, NADP, NADH, NADPH, H_2_O, NH_3_, phosphate, and pyrophosphate [3]. Finally, we linked the network distance to gene pairs by using the information on gene-reaction associations (see also above). We did not consider gene pairs that encode subunits of the same protein complex, since network distance is defined between reaction pairs.

### Operonic organization of E. coli genes.

Information on the operonic organization of E. coli genes was obtained from regulonDB [33]. Operons illustrate a strong functional interaction between genes, and it represents one mode of transcriptional co-regulation by precise gene co-expression.

### Transcription factor binding and gene-expression similarity.

Transcription factor (TF) binding sites upstream of E. coli operons were obtained from a previous study on gene regulation networks [21], which we updated with the recent interaction data from regulonDB. To reduce the number of possible incorrect TF–operon interactions, we did not include interactions from regulonDB that were solely based on microarray data [21].

We examined TF similarity between operon pairs to compare stringency in transcription factor regulation between flux (un)coupled genes. TF similarity is a measure of overlap in the set of bound TFs between operons and is defined as the total number of shared TFs between *two* operons divided by the total number of unique TFs regulating the two operons. For example, if TF x and y regulate operon 1 and TF x and z operon 2, the TF similarity will be 1/3. As TF regulation is a property of operons, we exclusively studied flux coupling on the level of operons. We determined TF similarity of operon pairs only once irrespective of the total number of flux coupled gene pairs belonging to the same operon pair.

Additionally, we compared the extent of co-expression between E. coli gene pairs for the same set of operon pairs that were studied for TF similarity. We obtained microarray data for E. coli from a recently constructed dataset [24]. In a similar way, we investigated mRNA-level co-expression of metabolic genes in S. cerevisiae by analyzing a large set of microarray data [2] . We established expression similarity (i.e., measure of co-expression) between genes by calculating Pearson correlation coefficients of the normalized log-ratios across microarray experiments.

### Statistical analyses.

Frequency tables were analyzed by chi-square tests to test the hypothesis of independency between factors. Moreover, we applied one- and two-way robust analysis of variance (ANOVA) and multiple pairwise comparison techniques developed by Rand Wilcox to avoid problems from non-normal distributions and heteroscedasticity [27]. The methods are based on Welch's statistics and the analysis of 20% trimmed means to increase the control over type I errors (i.e., rejecting null hypothesis when it is actually correct). We applied one- and two-way robust ANOVA using the “t1way” and “t2way” R functions, respectively. Multiple pairwise comparisons between variables (also called linear contrasts) were performed by using the “lincon” R function. Confidence intervals in related graphical representations were calculated by the “trimci” R function. All R functions can be found at http://www-rcf.usc.edu/∼rwilcox/.

Although we used Wilcox robust ANOVA throughout the article due to heteroscedasticities in our datasets, similar conclusions were drawn when conventional ANOVA was employed (unpublished data).

## Supporting Information

Dataset S1 List of Flux Coupled Gene Pairs (Sheet 1) Flux coupled gene pairs of *E.coli* (with gene duplicates/isoenzymes).
(Sheet 2) Flux coupled gene pairs of S. cerevisiae (with gene duplicates/isoenzymes).
(496 KB XLS)

Figure S1 Dependence of Network Distance and Co-Expression on the Type of Flux Coupling in E. coli Metabolism (A) Uncoupled gene pairs have higher network distances than flux coupled pairs (i.e., fully and directionally) in the metabolic network of E. coli (Wilcox robust one-way ANOVA, *p* ≈ 0), but fully and directionally coupled gene pairs do not differ in terms of overall network distance (Wilcox robust one-way ANOVA, *p* = 0.9).
(B) mRNA-level co-expression correlates with the type of flux coupling in the metabolic network of E. coli (Wilcox robust one-way ANOVA, *p* < 10^−14^).
(71 KB PDF)

Table S1 The Association between Operonic Organization and Network Distance for Each Specific Type of Flux Coupling (19 KB DOC)

Table S2 The Frequency of Intra- and Inter-Operonic Fully Coupled Gene Pairs in Different Network Distance Groups The fraction of intra-operonic pairs decreases with network distance (χ^2^ = 27.3, d.f. = 2, *p* < 10^−5^).
(19 KB DOC)

Table S3 The Frequency of Intra- and Inter-Operonic Fully Coupled Gene Pairs within “Non-Limiting” Operons in Different Network Distance Groups Apparently, none of the 25 inter-operonic gene pairs at large network distances (i.e., ≥5) were in “non-limiting” operons; however, we still see an association between network distance and operonic organization for pairs at distance < 5 (χ^2^ = 11.9, d.f. = 1, *p* < 10^−3^).
(19 KB DOC)

Table S4 The Association between Operonic Organization and Network Distance for Each Specific Type of Flux Coupling in E. coli
Network distance was calculated by assuming that all reactions are reversible. Because the average network distance is now 3.2, we categorized gene pairs into the following three network distance groups: i) distance 1; ii) distance 2; and iii) distance ≥3.
(20 KB DOC)

Table S5 The Association between Operonic Organization and Flux Coupling for Each Network Distance Group in E. coli
Network distance was calculated by assuming that all reactions are reversible. Because the average network distance is now 3.2, we categorized gene pairs into the following three network distance groups: i) distance 1; ii) distance 2; and iii) distance ≥3.
(20 KB DOC)

Table S6 The Effect of Flux Coupling and Network Distance on Co-Expression for E. coli
Network distance was calculated by assuming that all reactions are reversible. Because the average network distance is now 3.2, we categorized gene pairs into the following three network distance groups: i) distance 1; ii) distance 2; and iii) distance ≥3. Relative variance components (i.e., the fraction of total variance in co-expression explained by coupling and distance) were estimated by a general linear model where both flux coupling and distance were treated as random effects in an unbalanced factorial ANOVA design. Both expected means squares and maximum likelihood estimation were used for the estimation (Statistica 6.0, Statsoft).
(21 KB DOC)

Table S7 The Effect of Flux Coupling and Network Distance on Co-Expression for S. cerevisiae
Network distance was calculated by assuming that all reactions are reversible. Because the average network distance is now 3.8, we categorized gene pairs into the following three network distance groups: i) distance 1; ii) distance 2, 3; and iii) distance ≥4. Relative variance components (i.e., the fraction of total variance in co-expression explained by coupling and distance) were estimated by a general linear model where both flux coupling and distance were treated as random effects in an unbalanced factorial ANOVA design. Both expected means squares and maximum likelihood estimation were used for the estimation (Statistica 6.0, Statsoft).
(20 KB DOC)
