# Supplementary material for: Co-Regulation of Metabolic Genes Is Better Explained by Flux Coupling Than by Network Distance
Source: PLoS Comput Biol. 2008 Jan 25;4(1):e26. doi: 10.1371/journal.pcbi.0040026 (PMC2211535; doi:10.1371/journal.pcbi.0040026)
Supplement: Supplementary file 1 [file pcbi.0040026.sg001.pdf]

Supporting figures

Figure S1A Uncoupled gene pairs have higher network distances than flux coupled pairs (i.e. fully and directionally) in the metabolic network of *E. coli* (Wilcox robust one-way ANOVA, $p \approx 0$), but fully and directionally coupled gene pairs do not differ in terms of overall network distance (Wilcox robust one-way ANOVA, $p = 0.9$).

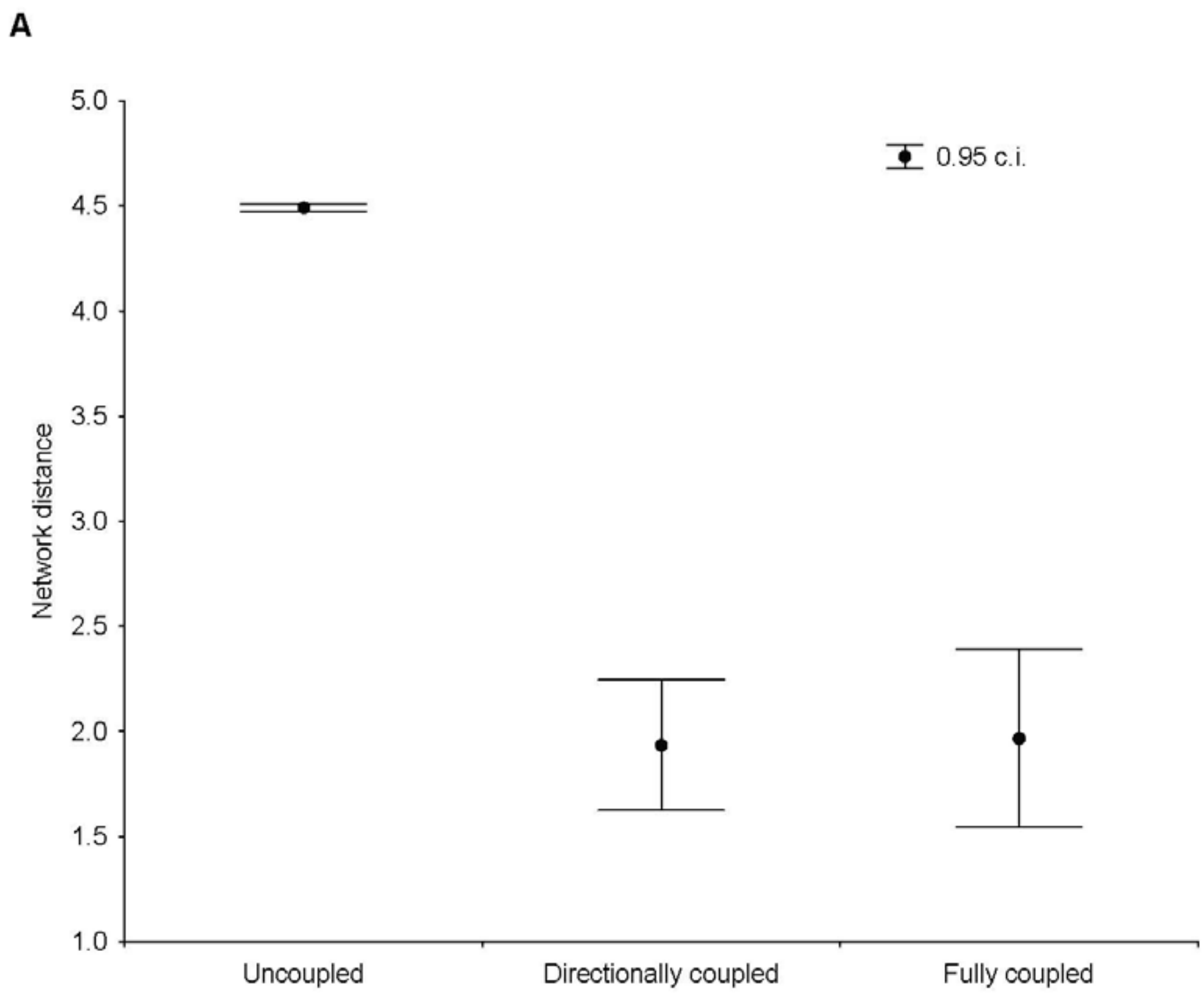

Figure S1B mRNA-level co-expression correlates with the type of flux coupling in the metabolic network of *E. coli* (Wilcox robust one-way ANOVA, $p < 10^{-14}$).

B

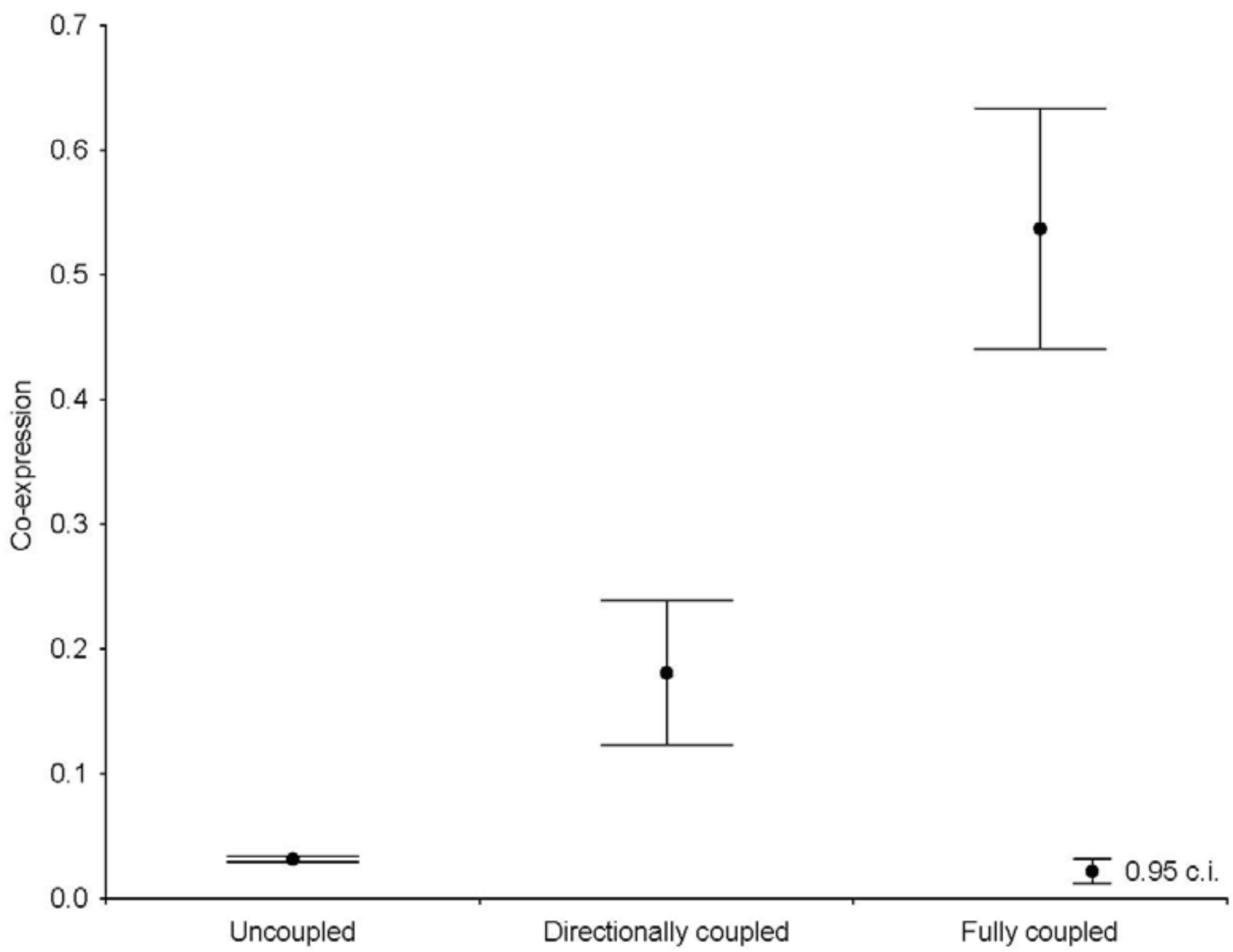

0.7
0.6
0.5
0.4
0.3
0.2
0.1
0.0
Co-expression
0.95 c.i.
Uncoupled
Directionally coupled
Fully coupled
