# Supplementary material for: Co-Regulation of Metabolic Genes Is Better Explained by Flux Coupling Than by Network Distance
Source: PLoS Comput Biol. 2008 Jan 25;4(1):e26. doi: 10.1371/journal.pcbi.0040026 (PMC2211535; doi:10.1371/journal.pcbi.0040026)
Supplement: Supplementary file 2 [file pcbi.0040026.st001.doc]

**Table S1** The association between operonic organization and network distance for each specific type of flux coupling.

|  | fully coupled pairs | directionally coupled pairs | uncoupled pairs |
| --- | --- | --- | --- |
| χ^2^-test | χ^2^ = 27.3  d.f. = 2  p < 10^-5^ | χ^2^ = 3.8  d.f. = 2  p=0.15 | χ^2^ = 476.6  d.f. = 2  p < 10^-103^ |
| Cramer’s V | 0.26 | (there was no significant association) | 0.07 |
