# Supplementary material for: Co-Regulation of Metabolic Genes Is Better Explained by Flux Coupling Than by Network Distance
Source: PLoS Comput Biol. 2008 Jan 25;4(1):e26. doi: 10.1371/journal.pcbi.0040026 (PMC2211535; doi:10.1371/journal.pcbi.0040026)
Supplement: Supplementary file 3 [file pcbi.0040026.st002.doc]

**Table S2** The frequency of intra- and inter-operonic fully coupled gene pairs in different network distance groups. The fraction of intra-operonic pairs decreases with network distance (χ^2^ = 27.3, d.f. = 2, p < 10^-5^)

|  | d=1 | d= 2,3,4 | d>=5 |
| --- | --- | --- | --- |
| Intra | 74 | 43 | 6 |
| Inter | 86 | 156 | 25 |

(Fully coupled gene pairs are either arranged within the same operon, i.e. intra-operonic, or arranged within different operons, i.e. inter-operonic).
