# Supplementary material for: Co-Regulation of Metabolic Genes Is Better Explained by Flux Coupling Than by Network Distance
Source: PLoS Comput Biol. 2008 Jan 25;4(1):e26. doi: 10.1371/journal.pcbi.0040026 (PMC2211535; doi:10.1371/journal.pcbi.0040026)
Supplement: Supplementary file 4 [file pcbi.0040026.st003.doc]

**Table S3** The frequency of intra- and inter-operonic fully coupled gene pairs within ‘non-limiting’ operons in different network distance groups. Apparently, none of the 25 inter-operonic gene pairs at large network distances (i.e. >= 5) were in ‘non-limiting’ operons, however, we still see an association between network distance and operonic organization for pairs at distance < 5 (χ^2^ = 11.9, d.f. = 1, p < 10^-3^).

|  | d=1 | d= 2,3,4 | d>=5 |
| --- | --- | --- | --- |
| Intra | 74 | 41 | 6 |
| Inter | 31 | 48 | 0 |
