# Supplementary material for: Co-Regulation of Metabolic Genes Is Better Explained by Flux Coupling Than by Network Distance
Source: PLoS Comput Biol. 2008 Jan 25;4(1):e26. doi: 10.1371/journal.pcbi.0040026 (PMC2211535; doi:10.1371/journal.pcbi.0040026)
Supplement: Supplementary file 5 [file pcbi.0040026.st004.doc]

**Table S4** The association between operonic organization and network distance for each specific type of flux coupling in *E. coli*. Network distance was calculated by assuming that all reactions are reversible. Because the average network distance is now 3.2, we categorized gene pairs into the following three network distance groups: i) distance 1, ii) distance 2 and iii) distance ≥ 3.

|  | fully coupled pairs | directionally coupled pairs | uncoupled pairs |
| --- | --- | --- | --- |
| χ^2^-test | χ^2^ = 19.0  d.f. = 2  p < 10^-4^ | χ^2^ = 3.4  d.f. = 2  p = 0.18 | χ^2^ = 307.4  d.f. = 2  p < 10^-66^ |
| Cramer’s V | 0.22 | (there was no significant association) | 0.056 |
