# Supplementary material for: Co-Regulation of Metabolic Genes Is Better Explained by Flux Coupling Than by Network Distance
Source: PLoS Comput Biol. 2008 Jan 25;4(1):e26. doi: 10.1371/journal.pcbi.0040026 (PMC2211535; doi:10.1371/journal.pcbi.0040026)
Supplement: Supplementary file 6 [file pcbi.0040026.st005.doc]

**Table S5** The association between operonic organization and flux coupling for each network distance group in *E. coli*. Network distance was calculated by assuming that all reactions are reversible. Because the average network distance is now 3.2, we categorized gene pairs into the following three network distance groups: i) distance 1, ii) distance 2 and iii) distance ≥ 3.

|  | d = 1 | d = 2 | d ≥ 3 |
| --- | --- | --- | --- |
| χ^2^-test | χ^2^ = 1088.2  d.f. = 2  p<10^-236^ | χ^2^ = 2928.37  d.f. = 2  p = 0 | χ^2^ = 9956.0  d.f. = 2  p = 0 |
| Cramer’s V | 0.52 | 0.34 | 0.38 |
