# Supplementary material for: Co-Regulation of Metabolic Genes Is Better Explained by Flux Coupling Than by Network Distance
Source: PLoS Comput Biol. 2008 Jan 25;4(1):e26. doi: 10.1371/journal.pcbi.0040026 (PMC2211535; doi:10.1371/journal.pcbi.0040026)
Supplement: Supplementary file 7 [file pcbi.0040026.st006.doc]

**Table S6** The effect of flux coupling and network distance on co-expression for *E. coli.*

Network distance was calculated by assuming that all reactions are reversible. Because the average network distance is now 3.2, we categorized gene pairs into the following three network distance groups: i) distance 1, ii) distance 2 and iii) distance ≥ 3. Relative variance components (i.e. the fraction of total variance in co-expression explained by coupling and distance) were estimated by a general linear model where both flux coupling and distance were treated as random effects in an unbalanced factorial ANOVA design. Both expected means squares and maximum likelihood estimation were used for the estimation (Statistica 6.0 by Statsoft, Inc.).

|  | Coupling | Distance | Interaction term |
| --- | --- | --- | --- |
| Fraction of variance explained (expected means squares method) | 36.1% | 0.6% | 4.1% |
| Fraction of variance explained (maximum likelihood method) | 32.1% | - | 2% |
